# Editorial for the *Insects* Special Issue “Bee Conservation: Behavior, Health and Pollination Ecology”

**DOI:** 10.3390/insects17030324

**Published:** 2026-03-17

**Authors:** Kit S. Prendergast

**Affiliations:** 1 School of Molecular & Life Sciences, Curtin University, Bently, WA 6102, Australia; kitprendergast21@gmail.com; 2 Centre for Sustainable Agricultural Systems, University of Southern Queensland, Toowoomba, QLD 4350, Australia

We live in a time of bee decline, with ever-increasing publications drawing attention to reductions in bee populations, pollinator deficits, and increases in threatening processes known to impact bee populations, including habitat loss, climate change, agricultural intensification, urbanization, pesticides, pathogens and introduced plants and bees [1,2,3,4,5,6,7]. There remain large lacunas in our knowledge in how to tackle and address this pollinator decline that threatens species and ecosystems at large. Knowledge on the basic yet absolutely core aspects of species ecology such as their nesting, foraging and reproductive behavior has been unknown for many species. On the other hand, for many plants, we do not have any knowledge on which pollinator species visits them or their pollination requirements. The factors that lead to healthy bee populations, especially wild populations, are similarly under-represented in the literature. This Special Issue focused on these core research areas—bee behavior, bee health and foraging ecology. Fourteen articles comprised the Special Issue “Bee Conservation: Behavior, Health and Pollination Ecology”.

It is encouraging to see that this Special Issue includes predominantly papers from the Global South and Australasia—regions suffering from a pollinator crisis, yet underrepresented in the literature [8,9]. In this collection, we have one paper from research conducted in Australia, six from Asia (Taiwan, Thailand, China), one from Africa (Morocco), one from the United Arab Emirates, and two from South America (Columbia, Peru). Numerous papers focus on wild non-honey bees—important given that non-honey bees comprise the bulk of bee biodiversity [10]—yet most research on behavior, health and pollination has focused on this species [11]. Prendergast and Wilson [12] even found that higher densities of this introduced species reduce nesting occupancy and the number of native bees observed in post-fire landscapes.

The article “Bee Hotels as a Tool for Post-Fire Recovery of Cavity-Nesting Native Bees” [12], which made the cover of *Insects* Volume 16, Issue 7 (July 2025), documented a world-first trial using artificial nesting substrates in the form of two types of bee hotels to evaluate and aid in the recolonization of native cavity-nesting bees after wildfires. Wildfires are anticipated to increase in intensity and severity under climate change [13], with cavity-nesting bees expected to be relatively more vulnerable [14]. Following installing 500 wooden block and 500 bamboo bee hotels at sites that had been burnt two years prior during the 2019/2020 Black Summer bushfires, it was found by the end of the monitoring period that over 900 nests were occupied, and over 800 of these were occupied by native bees. Moreover, a greater number of native bees were observed foraging at sites where bee hotels were installed compared with three burnt sites where they were not. This study provided a proof-of-concept that appropriately designed bee hotels can aid in recolonization.

Six papers in the Special Issue investigated pollination services, pollinator behavior, and plant–pollinator networks. Bipartite networks comprising plants and their pollinators can shed light on the health of pollination networks in different habitat types, and identify important plants to sustain pollinators [15,16,17]. In Peters and Cardona [18], the authors used ecological network science to provide recommendations to farmers on how to manage diverse agroforests to maximize their potential to support wild bees. This research found that bee abundance increased with flowering plant richness, whilst diverse agroforests with higher numbers of tree species supported less connected, less nested, and more specialized and modular bee–plant networks. They were also able to identify key plant species with the strongest impacts on network structure and stability, including those important for both whole bee as well as just the Meliponini bee assemblages. Cities are expanding worldwide, but they have the potential to harbor diverse pollinators, which are in turn important for urban agriculture and ecosystem functioning [19]. Results presented in this Special Issue by Chang et al. [20] in Hanzhong City found that 80 pollinator species occur, dominated by Lepidoptera, and that diversity indices were lowest in farmland than forest and agroforestry habitats. Looking at plant–pollinator networks, there was a relatively low diversity of plants visited by insects—55 compared with 58 visitors—and the network structure suggested a simplified, generalized network, with high nestedness, high generalization, and high connectedness. One of the most visited crop species was *Brassica napus*. Based on the gross agricultural product, yield of major pollinated crops, market price, and pollination dependence of Hanzhong City in 2023, the pollination service value of 11 major pollinated crops in Hanzhong City was estimated at CNY 48.8–352.9 million, 10.02–13.87% of the City’s agricultural output value, with fruit and vegetable crops having the highest pollination service value. This paper shows how a holistic study using detailed pollinator surveys, consideration of habitat type, plant preference, networks and pollination dependence can reveal an urban area’s pollinator community and provide incentive and recommendations to preserve it. The research by Bencharki et al. [21] also shows the importance of non-*Apis* bees, as well as exploring how different farming practices and landscape context influence alternative pollinators and how this translates into farmer income. The authors compared “Farming with Alternative Pollinators” (FAP), which uses marketable habitat enhancement plants, compared with monoculture fields in Morocco faba bean fields, and found that FAP fields had a higher diversity and abundance of pollinators and natural enemies, but the difference was less in large fields, as was the difference in net income (108% vs. 36% in small vs. large fields), which nevertheless was still significant. Farmer questionnaires also recognized the value of marketable habitat enhancement plants as a cost-effective, multi-benefit solution. The study reveals that whilst FAP has greater benefits at small scales, it is an effective solution for both biodiversity and farmers. Li et al. [22] investigated the relative contribution of wild pollinators to *Camellia oleifera*—a vital woody oil crop in China. The four main visitors were *Colletes gigas*, *Apis cerana*, *Vespa velutina*, and *Vespa soror*, and the solitary ground-nesting bee, *Colletes gigas*, was the most effective pollen vector, related to this species having significantly longer and denser scopae. This study shows how non-*Apis* bees can be superior pollinators, as well as highlighting how morphological features can be used to screen visitors for their potential as pollinating agents. Behavior can also influence the pollination effectiveness of different pollinating agents. Using an experimental approach, Hernández et al. [23] investigated how honey bee foraging decisions are shaped by floral trait distinctiveness and perceptions of gains and losses. They found that foragers developed flower fidelity to the color, offering higher nectar concentrations in each experiment, and flower color fidelity was higher when flower colors were more distinct, yet this also made it more difficult for bees to abandon the flower color in the reversal learning phase, revealing cognitive costs in adaptive foraging especially under changing floral landscapes. The research found that the honey bees used and integrated information from different axes of information: distinctiveness of color cues, magnitude of reward difference, and directionality (being stronger for losses than gains). Also on the topic of behavior, Huang et al. [24] found that for Cherry (*Prunus pseudocerasus* ‘Hongdeng’) in Northern China, the introduced *Bombus terrestris* had longer foraging hours and a bimodal foraging peak, as well as greater pollen-carrying capacity than the native *Apis cerana*. There was, however, no different yield or sale value between these two pollinator species, and the authors cautioned against widespread adoption of the introduced *Bombus terrestris* given its potential to cause ecological impacts on native ecosystems. Given China is a hotspot for *Bombus* diversity, the results of this study suggest a need to invest in supporting and investigating the pollinator potential of China’s indigenous bumble bees [25,26].

Documenting the distribution and diversity of native bees is critical for biodiversity assessments and conservation, and thus taxonomic papers are extremely valuable [27,28,29,30]. An important contribution to this Special Issue was a paper by Rasmussen and Sánchez [31]: “Taxonomic Impediment for Conservation: The Case of Bees in an Undersampled Tropical Mid-Elevation Site, San Martín, Peru”. The researchers conducted the first field survey ever of the bee fauna in any part of Peru, and found an incredible diversity of bees—181 species! Underscoring the taxonomic impediment in this part of the world, 44% of these species were undescribed. Their survey also provided a critical baseline for inventorying, as well as region-specific community compositions including flower and nesting associations on the basis of related species. Another important contribution to this Special Issue was a taxonomic paper on cleptoparasitic bees of the subgenus *Stelis* (*Stelidomorpha*) by Kasparek et al. [32]. Using both morphometric and genetic approaches, two new species, *Stelis alainensis* Kasparek sp. nov. and *Stelis surica* Kasparek sp. nov., both discovered in Oman and the United Arab Emirates, were described. One previously recognized subspecies, *Stelis aegyptiaca* ssp. *canaria* Warncke, 1992 was elevated to species rank as *S. canaria* Warncke, 1992. Additionally, *Stelis nasuta* (Latreille, 1809) was found to comprise three genetically distinct lineages with discrete geographic distributions, yet multivariate analyses of 11 morphometric traits revealed no consistent diagnostic differences, revealing the complicated nature of genetics vs. morphology when it comes to species delineations, and as such, these lineages were treated as phylospecies rather than formal taxa.

Disease is a known factor threatening bee populations, especially managed bees, and thus there is a need to better understand pathogen dynamics, as well as investigate ways to reduce pathogen exposure and improve bee health. Five papers in this Special Issue were on bee health. This included a review [33] which identified how plant products do not just provide bees with nutrition (pollen and nectar) and building materials (resin), but these plant products also have medicinal benefits to wild bees. Whilst 51 compounds were found to have ‘medicinal’ benefits in the pollen, nectar, and/or resin of over 200 species from 64 families, very few of these had been directly tested on bees or their disease agents. With important potential for plant compounds to help bees fight diseases and combat anthropogenic stressors, more research is required, and Morrison, Newburn and Fitch [33] set out an agenda to fill these gaps. The remaining four papers were studies on *Apis* species. Lertlakkanawat et al. [34] investigated the possibility of using longan syrup as a dietary supplement for honey bees and found that contrary to expectations, longan syrup actually reduced honey bee survivorship and just feeding sucrose solution resulted in better survivorship! Nevertheless, analyses of honey bee gut microbiomes found that longan syrup supplementation increased fermentative microorganisms such as *Lactobacillus* and *Fructobacillus*, which have known benefits for honey bee health, so there may be potential for low concentrations of logan syrup to have health benefits under certain contexts. It is important for such negative results to be published. In a world full of snake-oil remedies for bee declines, knowing what works and what does not is important. Two papers were on the honey bee pathogen *Vairimorpha ceranae*, which interferes with immune defenses and alters behavior. Li et al. [35] found that across a year, the prevalence or activity of *V. ceranae* increased with temperature, which does not bode well under projections for continuing increases in climate change, and represents another example of how anthropogenic climate change is adversely impacting bees [36]. Li, Chen, Chang, Wu and Nai [35] also found that applying probiotic, using lactic acid bacterium *Leuconostoc mesenteroides* TBE-8, had potential to treat microsporidiosis. [37] evaluated immune gene expression and locomotor behavior across five *Apis mellifera* subspecies (Carniolan, Caucasian, Syrian, Muğla ecotype, and Yığılca ecotype) following controlled *Vairimorpha ceranae* infection, revealing a large variation among these subspecies and that genetics matters, also pointing to the potential for artificial selection to improve bee health. Caucasian, Carniolan, and Yığılca showed strong immune gene activation, suggesting a more effective defense, whilst Syrian and Muğla bees had weaker immune responses and higher parasite levels, indicating greater vulnerability. Locomotor analysis revealed subspecies-specific behavioral responses, with suggestions of physiological trade-offs as well as alternative mechanisms other than gene activation to fight infection depending on subspecies. Environmental pollutants are of major concern to bee health, as anthropogenic activities continue to leach toxins into the air, soil and water [38,39]. Cadmium is one such pollutant of concern. In Li et al. [40], they found that with low levels of Cd in the soil, the amount of Cd in the pollen was negatively related to the body mass of the larvae, pupae, and worker bees, whilst nectar quantity governed foraging behavior, whilst at middle to high exposure levels, Cd concentrations in pollen and honey had adverse impacts on honey bee development and foraging behavior.

The papers in this Special Issue together highlight that understanding bee behavior, including nesting and foraging behavior, can help guide our ability to better improve bee health; conserve them in the face of threats like urbanization, agricultural expansion, fire and climate change; and also highlight the value of biodiversity surveying and good taxonomy, whilst underscoring that much remains to be known, especially when it comes to taxonomy.

## Data Availability

No new data were created or analyzed in this study. Data sharing is not applicable to this article.
